# Safety profile of bivalirudin in Chinese female patients undergoing percutaneous coronary intervention: a multi-center study

**DOI:** 10.1186/s12872-022-02474-3

**Published:** 2022-02-17

**Authors:** Fan Wu, Xueying Liu, Huazhong Ran, Qiwei Tang, Cheng Zhong, Yanqing Wu, Jun Xiao

**Affiliations:** 1 Department of Cardiovascular Medicine II, Xuchang Central Hospital, 30 Huatuo Road, Xuchang, 461001 Henan Province China; 2 Department of Cardiology, The First Hospital of Zhangjiakou, No.6 Chapel Lane, Xinhua Front Street, Qiaoxi District, Zhangjiakou, 075061 Hunan China; 3 Department of Cardio-Thoracic Surgery, ZhouKou Center Hospistal of Henan Province, East Section of Renmin Road, Zhoukou, 466699 Henan China; 4 Department of Cardiology, The First Affiliated Hospital of Henan University, 357 Ximen Dajie, Kaifeng, 475004 Henan China; 5 Department of Cardiovascular Medicine, Zhejiang Lvcheng Cardiovascular Hospital, 409 Gudun Road, Wenxin Street, Xihu District, Hangzhou, 310011 Zhejiang China; 6 Department of Cardiovascular Medicine, The Second Affiliated Hospital of Nanchang University, 1 Minde Road, Donghu District, Nanchang, 330006 China; 7 Department of Cardiovascular Medicine, Chongqing University Central Hospital, No. 1 Jiankang Road, Yuzhong District, Chongqing, 400014 China

**Keywords:** Bivalirudin, Percutaneous coronary intervention, Female, Adverse events and adverse drug reactions, Thrombocytopenia and bleeding

## Abstract

**Background:**

The present study aimed to comprehensively investigate the occurrence and risk factors of adverse events (AEs) or adverse drug reactions (ADRs) (especially for thrombocytopenia and bleeding) in Chinese female patients receiving bivalirudin during percutaneous coronary intervention (PCI).

**Methods:**

A total of 918 female patients from 27 Chinese medical centers took bivalirudin as anticoagulant for PCI were enrolled in this prospective, multi-center, intensive monitoring study. Safety data (AEs, ADRs, thrombocytopenia and bleeding) were collected from admission to 72 h post bivalirudin administration; then, patients were followed up at the 30^th^ day with the safety data collected as well.

**Results:**

One hundred and twenty (13.1%) patients occurred AEs, among which 7 (0.8%) cases experienced severe AEs, and 2 (0.2%) cases died. Besides, 40 (4.4%) patients occurred bivalirudin-related ADRs, in which 3 (0.3%) cases experienced severe ADRs, but 0 (0.0%) cases died. It was of note that 27 (2.9%) and 13 (1.4%) patients experienced thrombocytopenia and bleeding, respectively. Subsequent multivariate analyses observed that: clinical presentation of spontaneous coronary artery dissection (SCAD) (odds ratio (OR) = 3.191, *P* = 0.004), CRUSADE high risk (OR = 2.075, *P* = 0.031), multiple culprit vessel (OR = 2.328, *P* = 0.019) independently correlated with higher risk of bivalirudin-related ADRs; clinical presentation of SCAD (OR = 4.388, *P* = 0.002) and multiple culprit vessel (OR = 2.974, *P* = 0.010) independently linked with raised thrombocytopenia risk; history of diabetes mellitus (OR = 5.227, *P* = 0.007) and CRUSADE high risk (OR = 4.475, *P* = 0.016) were independent factor related to elevated bleeding risk.

**Conclusion:**

Bivalirudin is well tolerated with low ADRs, thrombocytopenia and bleeding incidences in Chinese female patients undergoing PCI.

## Background

Since the introduction of percutaneous coronary intervention (PCI) with or without drug-eluting stents (DES), it has been widely used to treat coronary artery disease (CAD) with good efficacy and tolerant adverse reactions [1, 2]. Gender differences in CAD commonly exist in several aspects, such as coronary anatomy, risk factors, comorbidities, CAD pathophysiology, clinical presentation response to pharmacotherapy mainly due to sex hormone variations [3–5]; meanwhile, gender also affects outcomes in CAD patients after PCI [5, 6]. Therefore, it is necessary to dig more information about the PCI application in female CAD patients.

Bivalirudin, as a synthetic congener of the naturally occurring drug hirudin, conquers several shortcomings of traditional indirect thrombin inhibitor such as heparin [7–9]. As for clinical utility, several large-scale, randomized, controlled trials have demonstrated the superiority of bivalirudin over heparin with or without Glycoprotein (GP) IIb/IIIa inhibitor in CAD patients underwent PCI [9–14]. However, there are few reports in terms of the adverse events (AEs) or adverse drug reactions (ADRs) (especially thrombocytopenia and bleeding) of bivalirudin as anticoagulant during PCI in the specific female patients, not to mention the lack of data on bivalirudin in Chinese patients.

Therefore, the current prospective, multi-center, intensive monitoring study aimed to comprehensively investigate the occurrence and risk factors of AEs and ADRs (especially for thrombocytopenia and bleeding) in Chinese female patients receiving bivalirudin as an anticoagulant during PCI.

## Methods

### Patients

A total of 918 female patients’ data were abstracted from a prospective, multi-center, intensive monitoring study which enrolled 3049 patients who underwent PCI and received bivalirudin as anticoagulant in 27 Chinese medical centers, between July 2018 and June 2019, aiming to further evaluate the safety of bivalirudin in a wide range of population. These 918 patients were chosen based on the criterium of being females.

In detail, the inclusion criteria were: (1) underwent PCI or percutaneous coronary angioplasty (PTCA); (2) used bivalirudin as anticoagulant; (3) age over 18 years; (4) female patients; (5) understood the study content and voluntarily participated in the study. Patients without use of bivalirudin were excluded from the study.

The study was conducted in accordance with the Declaration of Helsinki, and was approved by the Ethics Committee of the Chongqing University Central Hospital and all patients provided the written informed consents.

### Collection of clinical data

The following clinical data were collected: (i) demographic characteristics; (ii) medial history; (iii) clinical presentation: unstable angina (UA); ST-segment elevation myocardial infarction (STEMI); non-ST-segment elevation myocardial infarction (NSTMI) and spontaneous coronary artery dissection (SCAD); (iv) CRUSADE score (Can Rapid Risk Stratification of Unstable Angina Patients Suppress Adverse Outcomes with Early Implementation of the ACC/AHA Guidelines- bleeding score[15]); (v) PCI characteristics; (vi) administration of bivalirudin (vii) combined with GP IIb/IIIa inhibitors; (viii) thrombolysis in myocardial infarction (TIMI) flow grade (pre-procedure) and TIMI flow grade (post-procedure).

### Collection of safety data

Safety data were collected from hospital admission to 72 h after completion of bivalirudin administration. In addition, patients were followed up at the 30^th^ day “in person”, and the data were also collected at that time. ADRs were classified using the Systematic Organ Classification (SOC) and Preferred Term (PT) from the International Conference on the Coordination of International Drug Registration (ICH) Medical Dictionary for Regulatory Activities (MedDRA) 23.0.

### Definitions

AEs were defined as any unfavorable and unintended sign (including an abnormal laboratory finding), symptom, or disease that is temporarily associated with the use of a medical treatment that may or may not be considered related to the medical treatment. ADRs were defined as the harmful reactions of qualified drugs which was irrelevant to the purpose of medication under normal usage and dosage. Severe adverse events (SAEs) and severe adverse drug reactions (SADRs) were defined as one of the following events: (i) resulting in death; (ii) life-threatening consequences; (iii) leading to carcinogenesis, teratogenesis and birth defects; (iv) resulting in significant or permanent human disability or organ function damage; (v) resulting in hospitalization or prolonged length of stay; (vi) leading to other important medical events, and if not treated, the above listed conditions may occur. The severity of AEs and ADRs was classified according to the following criteria: (i) mild: symptoms were transient and did not affect the patient's normal daily activities; (ii) moderate: symptoms were significant and affect the patient’s normal daily activities, but tolerable, which were not required discontinuation of medication; (iii) severe: symptoms were obvious, intolerable and affected the patient's normal daily activities, which were required discontinuation of medication. The bleeding was defined and graded in terms of Bleeding Academic Research Consortium (BARC) consensus classification criteria [16]. The thrombocytopenia was defined as blood platelet below 75 × 10^9^/L.

### Statistical analysis

SAS 9.4 (SAS Institute, Inc., Cary, North Carolina, USA) was applied to complete data analysis. Normally distributed continuous variable was presented as mean value ± standard deviation, and categorized variable was expressed as count (percentage). Summaries of all AEs were calculated based on cases. If a case suffered from the same AE repeatedly, the most severe AE was reported in the study. Univariate logistic regression analysis was carried out to assess the factors related to risk of ADRs, thrombocytopenia and bleeding events; then the covariates with *P* value less than 0.05 in the univariate logistic regression analysis were further selected to be included in multivariable logistic model analysis (method: enter, in the SPSS software). *P* value < 0.05 was considered statistically significant.

## Results

### Study flow

**Fig. 1:**
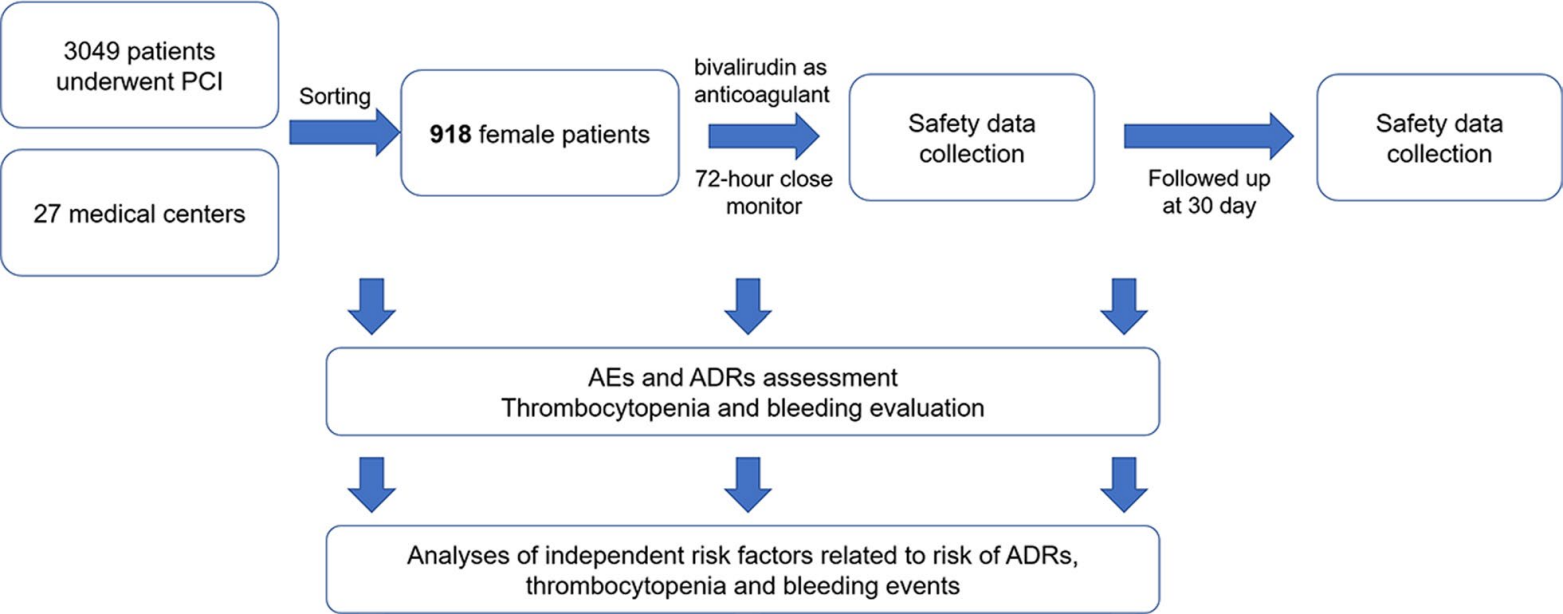
Study flow chart

Three thousand and forty-nine patients who underwent PCI and received bivalirudin as anticoagulant in 27 Chinese medical centers were initially enrolled, then 918 female patients were sorted out for the analysis in this current study (Fig. 1). Safety data collection was performed within 72-h close monitor and at 30^th^ day follow up. AEs, ADRs, thrombocytopenia and bleeding information, as well as their risk factors were evaluated.

### Patients’ characteristics

A total of 918 female patients receiving bivalirudin as an anticoagulant during PCI were enrolled with an age of 68.8 ± 9.2 years (Table 1). 360 (39.2%), 329 (35.8%), 129 (14.1%), 99 (10.8%) patients presented with unstable angina (UA), STEMI, NSTMI, and SCAD, respectively. Other detailed patients’ characteristics and PCI characteristics were exhibited in Table 1.

**Table 1:** Clinical characteristics of female patients

Items	Patients (N = 918)
Demographic characteristics	
Age (years), mean ± SD	68.8 ± 9.2
BMI (kg/m^2^), mean ± SD	24.5 ± 25.5
Medical history	
History of diabetes mellitus, No. (%)	275 (30.0)
History of allergy, No. (%)	105 (11.4)
History of cardiac surgery, No. (%)	72 (7.8)
History of renal function impairment, No. (%)	27 (2.9)
History of critical respiratory disease, No. (%)	20 (2.2)
Clinical presentation	
UA, No. (%)	360 (39.2)
STEMI, No. (%)	329 (35.8)
NSTMI, No. (%)	129 (14.1)
SCAD, No. (%)	99 (10.8)
Others, No. (%)	1 (0.1)
CRUSADE score	
Mean ± SD	35.4 ± 12.7
Risk stratification, No. (%)	
Very low risk (≤ 20)	81 (8.8)
Low risk (21 – 30)	275 (30.0)
Moderate risk (31– 40)	278 (30.3)
High risk (41 – 50)	158 (17.2)
Very high risk (> 50)	111 (12.1)
Unknown	15 (1.6)
PCI characteristics	
Operative timing, No. (%)	
Emergency operation	349 (38.0)
Elective operation	569 (62.0)
Types of coronary interventional therapy, No. (%)	
Stent implantation	872 (95.0)
Balloon dilatation	37 (4.0)
Thrombus aspiration	0 (0.0)
Others	9 (1.0)
Types of stents, No. (%)	
Drug stent	860 (93.7)
Bare stent	15 (1.6)
Unknown	43 (4.7)
Arterial access, No. (%)	
Brachial artery	1 (0.1)
Femoral artery	66 (7.2)
Radial artery	848 (92.4)
Others	3 (0.3)
Culprit vessel, No. (%)	
Single	715 (77.9)
Multiple	203 (22.1)
Administration of bivalirudin	
Preoperative or intraoperative, No. (%)	31 (3.4)
Postoperative ≤ 4 h, No. (%)	779 (84.9)
Postoperative > 4 h, No. (%)	108 (11.7)
Combined with GP IIb/IIIa inhibitors, No. (%)	663 (72.2)
TIMI flow grade (pre-procedure)	
0, No. (%)	241 (26.3)
1, No. (%)	145 (15.8)
2, No. (%)	86 (9.4)
3, No. (%)	442 (48.1)
Unknown, No. (%)	4 (0.4)
TIMI flow grade (post-procedure)	
0, No. (%)	4 (0.4)
1, No. (%)	4 (0.4)
2, No. (%)	14 (1.5)
3, No. (%)	895 (97.6)
Unknown, No. (%)	1 (0.1)

SD, standard deviation; BMI, body mass indexes; UA, unstable angina; STEMI, ST-segment elevation myocardial infarction; NSTMI, non-ST-segment elevation myocardial infarction; SCAD, spontaneous coronary artery dissection; CRUSADE, Can Rapid Risk Stratification of Unstable Angina Patients Suppress Adverse Outcomes with Early Implementation of the ACC/AHA Guidelines; PCI, percutaneous coronary intervention; GP, glycoprotein; TIMI, thrombolysis in myocardial infarction

### AEs, ADRs, thrombocytopenia and bleeding

One hundred and twenty (13.1%) patients occurred AEs, among which 7 (0.8%) cases experienced SAEs, and 2 (0.2%) cases died. In addition, 40 (4.4%) patients occurred bivalirudin-related ADRs, in which 3 (0.3%) cases experienced SADRs, but 0 (0.0%) cases died (Table 2). The detailed classifications of AEs and bivalirudin-related ADRs in SOC were presented in Table 3, which observed that gastrointestinal disorders and blood and lymphatic system disorders were the most common AEs and bivalirudin-related ADRs. In addition, it was noteworthy that 27 (2.9%) and 13 (1.4%) patients experienced thrombocytopenia and bleeding, respectively (Table 2).

**Table 2:** Summary of AEs and bivalirudin-related ADRs

Items	Incidence, No. (%)
Total AEs	120 (13.1)
SAEs	7 (0.8)
Hospitalization	4 (0.4)
Death	2 (0.2)
Other important medical events	1 (0.1)
Total bivalirudin-related ADRs	40 (4.4)
SADRs	3 (0.3)
Hospitalization	1 (0.1)
Death	0 (0.0)
Other important medical events	2 (0.2)
Thrombocytopenia	27 (2.9)
Bleeding	13 (1.4)

AEs, adverse events; ADRs, adverse drug reactions; SAEs, severe adverse events; SADRs, severe adverse drug reactions

**Table 3:** Detailed AEs and bivalirudin-related ADRs in System Organ Class (SOC)

Items	AEs, No. (%)				Bivalirudin-related ADRs, No. (%)			
	Total	Mild	Moderate	Severe	Total	Mild	Moderate	Severe
Total	120 (13.1)	110 (12.0)	4 (0.4)	6 (0.7)	40 (4.4)	38 (4.1)	2 (0.2)	0 (0.0)
Gastrointestinal disorders	38 (4.1)	35 (3.8)	2 (0.2)	1 (0.1)	10 (1.1)	8 (0.9)	2 (0.2)	0 (0.0)
Blood and lymphatic system disorders	28 (3.1)	28 (3.1)	0 (0.0)	0 (0.0)	28 (3.1)	28 (3.1)	0 (0.0)	0 (0.0)
General disorders and administration site conditions	27 (2.9)	27 (2.9)	0 (0.0)	0 (0.0)	0 (0.0)	0 (0.0)	0 (0.0)	0 (0.0)
Respiratory, thoracic, and mediastinal disorders	24 (2.6)	22 (2.4)	1 (0.1)	1 (0.1)	0 (0.0)	0 (0.0)	0 (0.0)	0 (0.0)
Nervous system disorders	15 (1.6)	11 (1.2)	2 (0.2)	2 (0.2)	0 (0.0)	0 (0.0)	0 (0.0)	0 (0.0)
Investigations	14 (1.5)	12 (1.3)	1 (0.1)	1 (0.1)	2 (0.2)	2 (0.2)	0 (0.0)	0 (0.0)
Cardiac disorders	12 (1.3)	10 (1.1)	0 (0.0)	2 (0.2)	1 (0.1)	1 (0.1)	0 (0.0)	0 (0.0)
Skin and subcutaneous tissue disorders	8 (0.9)	8 (0.9)	0 (0.0)	0 (0.0)	0 (0.0)	0 (0.0)	0 (0.0)	0 (0.0)
Renal and urinary disorders	7 (0.8)	7 (0.8)	0 (0.0)	0 (0.0)	0 (0.0)	0 (0.0)	0 (0.0)	0 (0.0)
Infections and infestations	6 (0.7)	6 (0.7)	0 (0.0)	0 (0.0)	0 (0.0)	0 (0.0)	0 (0.0)	0 (0.0)
Metabolism and nutrition disorders	5 (0.5)	5 (0.5)	0 (0.0)	0 (0.0)	0 (0.0)	0 (0.0)	0 (0.0)	0 (0.0)
Hepatobiliary disorders	5 (0.5)	5 (0.5)	0 (0.0)	0 (0.0)	0 (0.0)	0 (0.0)	0 (0.0)	0 (0.0)
Psychiatric disorders	5 (0.5)	5 (0.5)	0 (0.0)	0 (0.0)	0 (0.0)	0 (0.0)	0 (0.0)	0 (0.0)
Vascular disorders	4 (0.4)	4 (0.4)	0 (0.0)	0 (0.0)	0 (0.0)	0 (0.0)	0 (0.0)	0 (0.0)
Musculoskeletal and connective tissue disorders	3 (0.3)	3 (0.3)	0 (0.0)	0 (0.0)	0 (0.0)	0 (0.0)	0 (0.0)	0 (0.0)
Injury, poisoning and procedural complications	2 (0.2)	2 (0.2)	0 (0.0)	0 (0.0)	1 (0.1)	1 (0.1)	0 (0.0)	0 (0.0)
Immune system disorders	1 (0.1)	1 (0.1)	0 (0.0)	0 (0.0)	0 (0.0)	0 (0.0)	0 (0.0)	0 (0.0)

AEs, adverse events; ADRs, adverse drug reactions

### Factors related to bivalirudin-related ADRs risk

Univariate analyses showed that clinical presentation of UA was correlated with lower risk of bivalirudin-related ADRs (*P* = 0.006), whereas clinical presentation of SCAD (*P* = 0.001), CRUSADE high risk (*P* = 0.005), multiple culprit vessel (*P* = 0.048), preoperative or intraoperative administration of bivalirudin (*P* = 0.026) were associated with higher risk of bivalirudin-related ADRs. Subsequent multivariate analyses revealed that clinical manifestations of SCAD (*P* = 0.004), CRUSADE high risk (*P* = 0.031), multiple culprit vessel (*P* = 0.019) independently correlated with higher risk of bivalirudin-related ADRs (Table 4).

**Table 4:** Analysis of factors related to ADRs

Items	Bivalirudin-related ADRs		Univariate		Multivariate	
	No (%)	Yes (%)	*P* value	OR (95% CI)	*P* value	OR (95% CI)
Age			0.146		–	
> 75 years	217 (93.9)	14 (6.1)		1.640 (0.841–3.198)		–
≤ 75 years	661 (96.2)	26 (3.8)		Reference		–
BMI			0.284		–	
> 28 kg/m^2^	85 (97.7)	2 (2.3)		0.455 (0.108–1.920)		–
≤ 28 kg/m^2^	735 (95.1)	38 (4.9)		Reference		–
History of diabetes mellitus			0.159		–	
Yes	259 (94.2)	16 (5.8)		1.593 (0.833–3.049)		–
No	619 (96.3)	24 (3.7)		Reference		–
History of allergy			0.223		–	
Yes	98 (93.3)	7 (6.7)		1.688 (0.727–3.919)		–
No	780 (95.9)	33 (4.1)		Reference		–
History of cardiac surgery			0.268		–	
Yes	67 (93.1)	5 (6.9)		1.729 (0.656–4.560)		–
No	811 (95.9)	35 (4.1)		Reference		–
History of renal function impairment			0.095		–	
Yes	24 (88.9)	3 (11.1)		2.885 (0.831–10.015)		–
No	854 (95.8)	37 (4.2)		Reference		–
History of critical respiratory disease			0.887		–	
Yes	19 (95.0)	1 (5.0)		1.159 (0.151–8.883)		–
No	859 (95.7)	39 (4.3)		Reference		–
Clinical presentation-UA			**0.006**		0.088	
Yes	353 (98.1)	7 (1.9)		0.315 (0.138–0.721)		0.463 (0.191–1.122)
No	525 (94.1)	33 (5.9)		Reference		Reference
Clinical presentation-STEMI			0.371		–	
Yes	312 (94.8)	17 (5.2)		1.341 (0.706–2.548)		–
No	566 (96.1)	23 (3.9)		Reference		–
Clinical presentation–NSTMI			0.773		–	
Yes	124 (96.1)	5 (3.9)		0.869 (0.334–2.260)		–
No	754 (95.6)	35 (4.4)		Reference		–
Clinical presentation–SCAD			**0.001**		**0.004**	
Yes	88 (88.9)	11 (11.1)		3.405 (1.644–7.053)		3.191 (1.446–7.044)
No	790 (96.5)	29 (3.5)		Reference		Reference
CRUSADE risk stratification			**0.005**		**0.031**	
High risk	229 (92.3)	19 (7.7)		2.505 (1.323–4.744)		2.075 (1.070–4.024)
Non-high risk	634 (96.8)	21 (3.2)		Reference		Reference
Operative timing			0.209		–	
Elective operation	548 (96.3)	21 (3.7)		0.666 (0.353–1.256)		–
Emergency operation	330 (94.6)	19 (5.4)		Reference		–
Types of coronary interventional therapy			0.997		–	
Stent implantation	834 (95.6)	38 (4.4)		1.002 (0.234–4.290)		–
Others	44 (95.7)	2 (4.3)		Reference		–
Types of stents			0.659		–	
Drug stent	823 (95.7)	37 (4.3)		0.629 (0.081–4.915)		–
Others	14 (93.3)	1 (6.7)		Reference		–
Arterial access			0.564		–	
Radial artery	812 (95.8)	36 (4.2)		0.732 (0.253–2.118)		–
Others	66 (94.3)	4 (5.7)		Reference		–
Culprit vessel			**0.048**		**0.019**	
Multiple	189 (93.1)	14 (6.9)		1.963 (1.005–3.834)		2.328 (1.146–4.728)
Single	689 (96.4)	26 (3.6)		Reference		Reference
Administration of bivalirudin-preoperative or intraoperative			**0.026**		0.116	
Yes	27 (87.1)	4 (12.9)		3.502 (1.164–10.539)		2.522 (0.796–7.990)
No	851 (95.9)	36 (4.1)		Reference		Reference
Administration of bivalirudin-postoperative ≤ 4 h			0.383		–	
Yes	747 (95.9)	32 (4.1)		0.701 (0.316–1.556)		–
No	131 (94.2)	8 (5.8)		Reference		–
Administration of bivalirudin-postoperative > 4 h			0.724		–	
Yes	104 (96.3)	4 (3.7)		0.827 (0.288–2.370)		–
No	774 (95.6)	36 (4.4)		Reference		–
Combined with GP IIb/IIIa inhibitors			0.265		–	
Yes	631 (95.2)	32 (4.8)		1.566 (0.712–3.445)		–
No	247 (96.9)	8 (3.1)		Reference		–
TIMI flow grade (pre-procedure)			0.491		–	
2–3	507 (96.0)	21 (4.0)		0.800 (0.424–1.510)		–
0–1	367 (95.1)	19 (4.9)		Reference		–
TIMI flow grade (post-procedure)			0.284		–	
2–3	870 (95.7)	39 (4.3)		0.314 (0.038–2.613)		–
0–1	7 (87.5)	1 (12.5)		Reference		–

Bold value means statistically significant

ADRs, adverse drug reactions; OR, odds ratio; CI, confidence interval; BMI, body mass indexes; UA, unstable angina; STEMI, ST-segment elevation myocardial infarction; NSTMI, non-ST-segment elevation myocardial infarction; SCAD, spontaneous coronary artery dissection; CRUSADE, Can Rapid Risk Stratification of Unstable Angina Patients Suppress Adverse Outcomes with Early Implementation of the ACC/AHA Guidelines; GP, glycoprotein; TIMI, thrombolysis in myocardial infarction

### Factors related to thrombocytopenia and bleeding risk

Univariate analyses observed that clinical presentation of UA was associated with reduced thrombocytopenia risk (*P* = 0.032), whereas clinical presentation of SCAD (*P* < 0.001), multiple culprit vessel (*P* = 0.022) and preoperative or intraoperative administration of bivalirudin (*P* = 0.036) were associated with an increased thrombocytopenia risk. After adjustment for multivariate analysis, only clinical presentation of SCAD (*P* = 0.002) and multiple culprit vessel (*P* = 0.010) were independently correlated with higher thrombocytopenia risk (Table 5).

**Table 5:** Analysis of factors related to thrombocytopenia

Items	Thrombocytopenia		Univariate		Multivariate	
	No (%)	Yes (%)	*P* value	OR (95% CI)	*P* value	OR (95% CI)
Age			0.324		–	
> 75 years	222 (96.1)	9 (3.9)		1.507 (0.667–3.402)		–
≤ 75 years	669 (97.4)	18 (2.6)		Reference		–
BMI			0.285		–	
> 28 kg/m^2^	86 (98.9)	1 (1.1)		0.334 (0.045–2.493)		–
≤ 28 kg/m^2^	747 (96.6)	26 (3.4)		Reference		–
History of diabetes mellitus			0.970		–	
Yes	267 (97.1)	8 (2.9)		0.984 (0.425–2.276)		–
No	624 (97.0)	19 (3.0)		Reference		–
History of allergy			0.247		–	
Yes	100 (95.2)	5 (4.8)		1.798 (0.666–4.853)		–
No	791 (97.3)	22 (2.7)		Reference		–
History of cardiac surgery			0.181		–	
Yes	68 (94.4)	4 (5.6)		2.105 (0.708–6.262)		–
No	823 (97.3)	23 (2.7)		Reference		–
History of renal function impairment			0.181		–	
Yes	25 (92.6)	2 (7.4)		2.771 (0.622–12.347)		–
No	866 (97.2)	25 (2.8)		Reference		–
History of critical respiratory disease			0.587		–	
Yes	19 (95.0)	1 (5.0)		1.765 (0.228–13.689)		–
No	872 (97.1)	26 (2.9)		Reference		–
Clinical presentation-UA			**0.032**		0.187	
Yes	355 (98.6)	5 (1.4)		0.343 (0.129–0.915)		0.492 (0.171–1.412)
No	536 (96.1)	22 (3.9)		Reference		Reference
Clinical presentation-STEMI			0.895		–	
Yes	319 (97.0)	10 (3.0)		1.055 (0.477–2.331)		–
No	572 (97.1)	17 (2.9)		Reference		–
Clinical presentation-NSTMI			0.656		–	
Yes	126 (97.7)	3 (2.3)		0.759 (0.225–2.558)		–
No	765 (97.0)	24 (3.0)		Reference		–
Clinical presentation-SCAD			** < 0.001**		**0.002**	
Yes	90 (90.9)	9 (9.1)		4.450 (1.942–10.198)		4.388 (1.754–10.981)
No	801 (97.8)	18 (2.2)		Reference		Reference
CRUSADE risk stratification			0.122		–	
High risk	237 (95.6)	11 (4.4)		1.854 (0.848–4.052)		–
No high risk	639 (97.6)	16 (2.4)		Reference		–
Operative timing			0.767		–	
Elective operation	553 (97.2)	16 (2.8)		0.889 (0.408–1.938)		–
Emergency operation	338 (96.8)	11 (3.2)		Reference		–
Types of coronary interventional therapy			0.665		–	
Stent implantation	846 (97.0)	26 (3.0)		0.672 (0.111–4.056)		–
Others	45 (97.8)	1 (2.2)		Reference		–
Types of stents			0.410		–	
Drug stent	835 (97.1)	25 (2.9)		0.419 (0.053–3.313)		–
Others	14 (93.3)	1 (6.7)		Reference		–
Arterial access			0.163		–	
Radial artery	825 (97.3)	23 (2.7)		0.460 (0.155–1.370)		–
Others	66 (94.3)	4 (5.7)		Reference		–
Culprit vessel			**0.022**		**0.010**	
Multiple	192 (94.6)	11 (5.4)		2.503 (1.143–5.483)		2.974 (1.302–6.792)
Single	699 (97.8)	16 (2.2)		Reference		Reference
Administration of bivalirudin-preoperative or intraoperative			**0.036**		0.081	
Yes	28 (90.3)	3 (9.7)		3.853 (1.095–13.553)		3.220 (0.867–11.953)
No	863 (97.3)	24 (2.7)		Reference		Reference
Administration of bivalirudin-postoperative ≤ 4 h			0.962		–	
Yes	756 (97.0)	23 (3.0)		1.027 (0.350–3.016)		–
No	135 (97.1)	4 (2.9)		Reference		–
Administration of bivalirudin-postoperative > 4 h			0.216		–	
Yes	107 (99.1)	1 (0.9)		0.282 (0.038–2.098)		–
No	784 (96.8)	26 (3.2)		Reference		–
Combined with GP IIb/IIIa inhibitors			0.281		–	
Yes	641 (96.7)	22 (3.3)		1.716 (0.643–4.581)		–
No	250 (98.0)	5 (2.0)		Reference		–
TIMI flow grade (pre–procedure)			0.074		–	
2–3	517 (97.9)	11 (2.1)		0.492 (0.226–1.072)		–
0–1	370 (95.9)	16 (4.1)		Reference		–
TIMI flow grade (post-procedure)			0.999		–	
2–3	882 (97.0)	27 (3.0)		–		–
0–1	8 (100.0)	0 (0.0)		–		–

Bold value means statistically significant

OR, odds ratio; CI, confidence interval; BMI, body mass indexes; UA, unstable angina; STEMI, ST-segment elevation myocardial infarction; NSTMI, non-ST-segment elevation myocardial infarction; SCAD, spontaneous coronary artery dissection; CRUSADE, Can Rapid Risk Stratification of Unstable Angina Patients Suppress Adverse Outcomes with Early Implementation of the ACC/AHA Guidelines; GP, glycoprotein; TIMI, thrombolysis in myocardial infarction

In terms of bleeding risk, univariate analyses showed that clinical presentation of UA (*P* = 0.048) and higher post-procedure TIMI flow grade (*P* = 0.033) were associated with a reduced risk of bleeding, but history of diabetes mellitus (*P* = 0.005) and CRUSADE high risk (*P* = 0.003) were linked to an increased risk of bleeding. Further multivariate analyses found that only history of diabetes mellitus (*P* = 0.007) and CRUSADE high risk (*P* = 0.016) were independent factor related to elevated risk of bleeding (Table 6).

**Table 6:** Analysis of factors related to bleeding

Items	Bleeding		Univariate		Multivariate	
	No (%)	Yes (%)	*P* value	OR (95% CI)	*P* value	OR (95% CI)
Age			0.273		–	
> 75 years	226 (97.8)	5 (2.2)		1.878 (0.608–5.798)		–
≤ 75 years	679 (98.8)	8 (1.2)		Reference		–
BMI			0.997		–	
> 28 kg/m^2^	87 (100.0)	0 (0.0)		–		–
≤ 28 kg/m^2^	760 (98.3)	13 (1.7)		–		–
History of diabetes mellitus			**0.005**		**0.007**	
Yes	266 (96.7)	9 (3.3)		5.405 (1.650–17.704)		5.227 (1.562–17.495)
No	639 (99.4)	4 (0.6)		Reference		Reference
History of allergy			0.654		–	
Yes	103 (98.1)	2 (1.9)		1.416 (0.309–6.476)		–
No	802 (98.6)	11 (1.4)		Reference		–
History of cardiac surgery			0.984		–	
Yes	71 (98.6)	1 (1.4)		0.979 (0.125–7.637)		–
No	834 (98.6)	12 (1.4)		Reference		–
History of renal function impairment			0.328		–	
Yes	26 (96.3)	1 (3.7)		2.817 (0.353–22.482)		–
No	879 (98.7)	12 (1.3)		Reference		–
History of critical respiratory disease			0.998		–	
Yes	20 (100.0)	0 (0.0)		–		–
No	885 (98.6)	13 (1.4)		–		–
Clinical presentation-UA			**0.048**		0.103	
Yes	359 (99.7)	1 (0.3)		0.127 (0.016–0.979)		0.178 (0.022–1.420)
No	546 (97.8)	12 (2.2)		Reference		Reference
Clinical presentation-STEMI			0.182		–	
Yes	322 (97.9)	7 (2.1)		2.112 (0.704–6.339)		–
No	583 (99.0)	6 (1.0)		Reference		–
Clinical presentation-NSTMI			0.889		–	
Yes	127 (98.4)	2 (1.6)		1.114 (0.244–5.084)		–
No	778 (98.6)	11 (1.4)		Reference		–
Clinical presentation-SCAD			0.164		–	
Yes	96 (97.0)	3 (3.0)		2.528 (0.684–9.346)		–
No	809 (98.8)	10 (1.2)		Reference		–
CRUSADE risk stratification			**0.003**		**0.016**	
High risk	239 (96.4)	9 (3.6)		6.129 (1.870–20.087)		4.475 (1.323–15.134)
No high risk	651 (99.4)	4 (0.6)		Reference		Reference
Operative timing			0.090		–	
Elective operation	564 (99.1)	5 (0.9)		0.378 (0.123–1.164)		–
Emergency operation	341 (97.7)	8 (2.3)		Reference		–
Types of coronary interventional therapy			0.658		–	
Stent implantation	860 (98.6)	12 (1.4)		0.628 (0.080–4.936)		–
Others	45 (97.8)	1 (2.2)		Reference		–
Types of stents			0.999		–	
Drug stent	848 (98.6)	12 (1.4)		–		–
Others	15 (100.0)	0 (0.0)		–		–
Arterial access			0.997		–	
Radial artery	835 (98.5)	13 (1.5)		–		–
Others	70 (100.0)	0 (0.0)		–		–
Culprit vessel			0.559		–	
Multiple	201 (99.0)	2 (1.0)		0.637 (0.140–2.896)		–
Single	704 (98.5)	11 (1.5)		Reference		–
Administration of bivalirudin-preoperative or intraoperative			0.401		–	
Yes	30 (96.8)	1 (3.2)		2.431 (0.306–19.304)		–
No	875 (98.6)	12 (1.4)		Reference		–
Administration of bivalirudin-postoperative ≤ 4 h			0.126		–	
Yes	770 (98.8)	9 (1.2)		0.394 (0.120–1.299)		–
No	135 (97.1)	4 (2.9)		Reference		–
Administration of bivalirudin-postoperative > 4 h			0.215		–	
Yes	105 (97.2)	3 (2.8)		2.286 (0.619–8.439)		–
No	800 (98.8)	10 (1.2)		Reference		–
Combined with GP IIb/IIIa inhibitors			0.326		–	
Yes	652 (98.3)	11 (1.7)		2.134 (0.470–9.696)		–
No	253 (99.2)	2 (0.8)		Reference		–
TIMI flow grade (pre-procedure)			0.173		–	
2–3	518 (98.1)	10 (1.9)		2.465 (0.674–9.016)		–
0–1	383 (99.2)	3 (0.8)		Reference		–
TIMI flow grade (post-procedure)			**0.033**		0.069	
2–3	897 (98.7)	12 (1.3)		0.094 (0.011–0.821)		0.105 (0.009–1.187)
0–1	7 (87.5)	1 (12.5)		Reference		Reference

Bold value means statistically significant

OR, odds ratio; CI, confidence interval; BMI, body mass indexes; UA, unstable angina; STEMI, ST-segment elevation myocardial infarction; NSTMI, non-ST-segment elevation myocardial infarction; SCAD, spontaneous coronary artery dissection; CRUSADE, Can Rapid Risk Stratification of Unstable Angina Patients Suppress Adverse Outcomes with Early Implementation of the ACC/AHA Guidelines; GP, glycoprotein; TIMI, thrombolysis in myocardial infarction

## Discussion

The efforts to investigate gender difference in CAD features or its treatment outcomes have never been stopped. For instance, it has been revealed that compared to male CAD patients, female CAD patients are often with older age at presentation, are accompanied with more comorbidities and severe disease condition [5, 17–23]. Furthermore, a growing number of researches observe that female patients underwent PCI exhibit a worse prognosis compared to male patients [24–29]. Notably, a recent meta-analysis analyzes 49 studies involving 1,032,828 patients reporting gender-specific outcomes in CAD patients underwent PCI, which discovers that major adverse cardiovascular event (MACE) and mortality are both increased, while revascularization rate is decreased in female patients compared to male patients [6]. Furthermore, it’s also disclosed that gender-specific effect exists regarding different antiplatelet strategies [30, 31]. For example, the effect of P2Y12 inhibitor monotherapy after coronary revascularisation differs between females and males [30]. These evidenced point out the emphasis of PCI treated female patients.

Several top-level trials have reported the preeminence of bivalirudin over conventional heparin in terms of adverse events [9–14], for instance: a previous trial observed that net adverse clinical events (NACEs) (9.2% vs. 12.1%) and major bleeding (4.9% vs. 8.3%) were both attenuated by bivalirudin monotherapy compared with unfractionated heparin (UFH) plus a GP IIb/IIIa inhibitor in patients undergoing PCI [12]; another trial discovers that bivalirudin with provisional GP IIb/IIIa inhibitor reduces major bleeding rate versus heparin with planned GP IIb/IIIa inhibitor (2.4% vs 4.1%) in patients during PCI [11]. However, the studies focusing on female patients in this field are still finite. A trial discloses that bivalirudin achieves reduced incidences of 30-day NACEs (6.3% vs. 21.5%), any bleeding (2.4% vs. 12.8%) and BARC 2–5 type bleeding (1.6% vs. 7.2%) compared to heparin with or without tirofiban in female patients undergoing PCI [32]. Nevertheless, there are limited reports regarding the AEs or ADRs of bivalirudin during PCI in real-world condition, not to mention that bivalirudin lacks data in Chinese female patients. In our present study, we observed that the incidence of AEs, SAEs, bivalirudin-related ADRs and bivalirudin-related SADRs was 13.1%, 0.8%, 4.4% and 0.3%, respectively, furthermore, 2.9% and 1.4% patients experienced thrombocytopenia and bleeding, in Chinese female patients undergoing PCI with bivalirudin as anticoagulant. Our data of AEs incidence was within the range of that in previous studies, which did not assess the bivalirudin-related ADRs incidence, therefore, it could not be referred. Interestingly, it was observed that thrombocytopenia and bleeding incidences by bivalirudin were relatively less in our present study compared to those published data previously, the possible explanations are: (1) Chinese patients may have less complications (such as obesity, hyperlipidemia, diabetes, kidney diseases), which is relates to less thrombocytopenia and bleeding risk; (2) Different study design, observational period and so on might influence the data.

Subsequently, in our study, it was found that clinical manifestation of SCAD, CRUSADE high risk, multiple culprit vessel was independently correlated with higher risk of bivalirudin-related ADRs. Several possible explanations are listed as follows: (1) PCI is selectively proposed for SCAD treatment with an increased risk of complications such as Iatrogenic dissection, acute vascular occlusion and hematoma extending, is therefore correlated with higher ADRs [33]; (2) CRUSADE high risk and diabetes mellitus are well-known risk factors for bleeding during PCI, therefore relates to increased ADRs and bleeding risk; (3) multiple culprit vessel indicates more severe disease conditions leading to higher ADRs.

Some limitations of the current study needed to be addressed: firstly, due to the total bivalirudin-related ADRs incidence was low, the sample size of nearly one thousand might not be sufficient to make a confirmative conclusion, therefore future larger sample-sized study was needed; secondly, the low ADRs, thrombocytopenia and bleeding incidences also reduced the statistical power of logistic analyses.

## Conclusion

To be conclusive, bivalirudin is well tolerated with low ADRs, thrombocytopenia and bleeding incidences in female patients undergoing PCI.

## Data Availability

All relevant data is presented in the manuscript and supporting material.

## Abbreviations

PCI: Percutaneous coronary intervention
DES: Drug-eluting stents
CAD: Coronary artery disease
AEs: Adverse events
ADRs: Adverse drug reactions;
BMI: Body mass indexes
UA: Unstable angina
STEMI: ST-segment elevation myocardial infarction
NSTMI: Non-ST-segment elevation myocardial infarction
SCAD: Spontaneous coronary artery dissection
GP: Glycoprotein
TIMI: Thrombolysis in myocardial infarction
SOC: Systematic organ classification
PT: Preferred term
MedDRA: Medical dictionary for regulatory activities
SAEs: Severe adverse events
SADRs: Severe adverse drug reactions
UA: Unstable angina
NACEs: Net adverse clinical events
UFH: Unfractionated heparin
